# Supplementary figures and images for: Nucleus softens during herpesvirus infection
Source: PLoS Pathog. 2026 Jan 20;22(1):e1013873. doi: 10.1371/journal.ppat.1013873 (PMC12818678; doi:10.1371/journal.ppat.1013873)

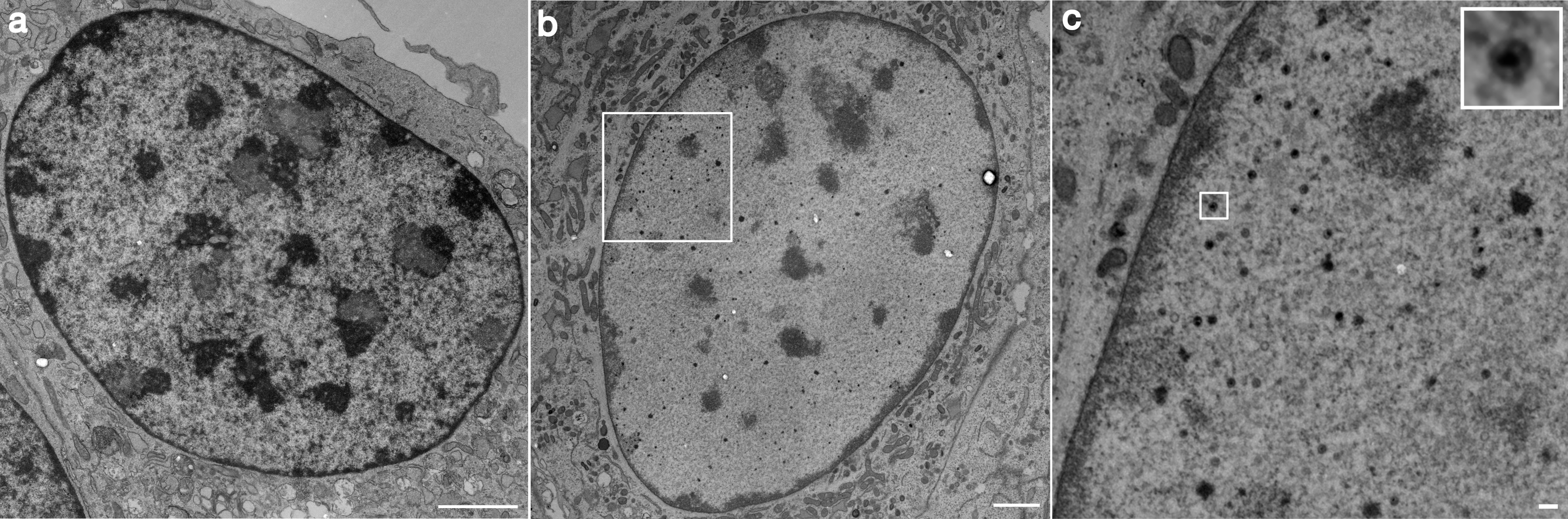

Supplement: S1 Fig — Representative transmission electron microscopy images of (A) a noninfected and (B) an infected MEF cell at 8 hpi. ChromEM labeling was used to enhance DNA contrast. (C) Full viral capsids with DNA staining are shown. White squares show the magnified nucleoplasm and a full capsid. Scale bars, 2 µm (A, B) and 0.2 µm (C). (TIF) [file ppat.1013873.s001.tif]

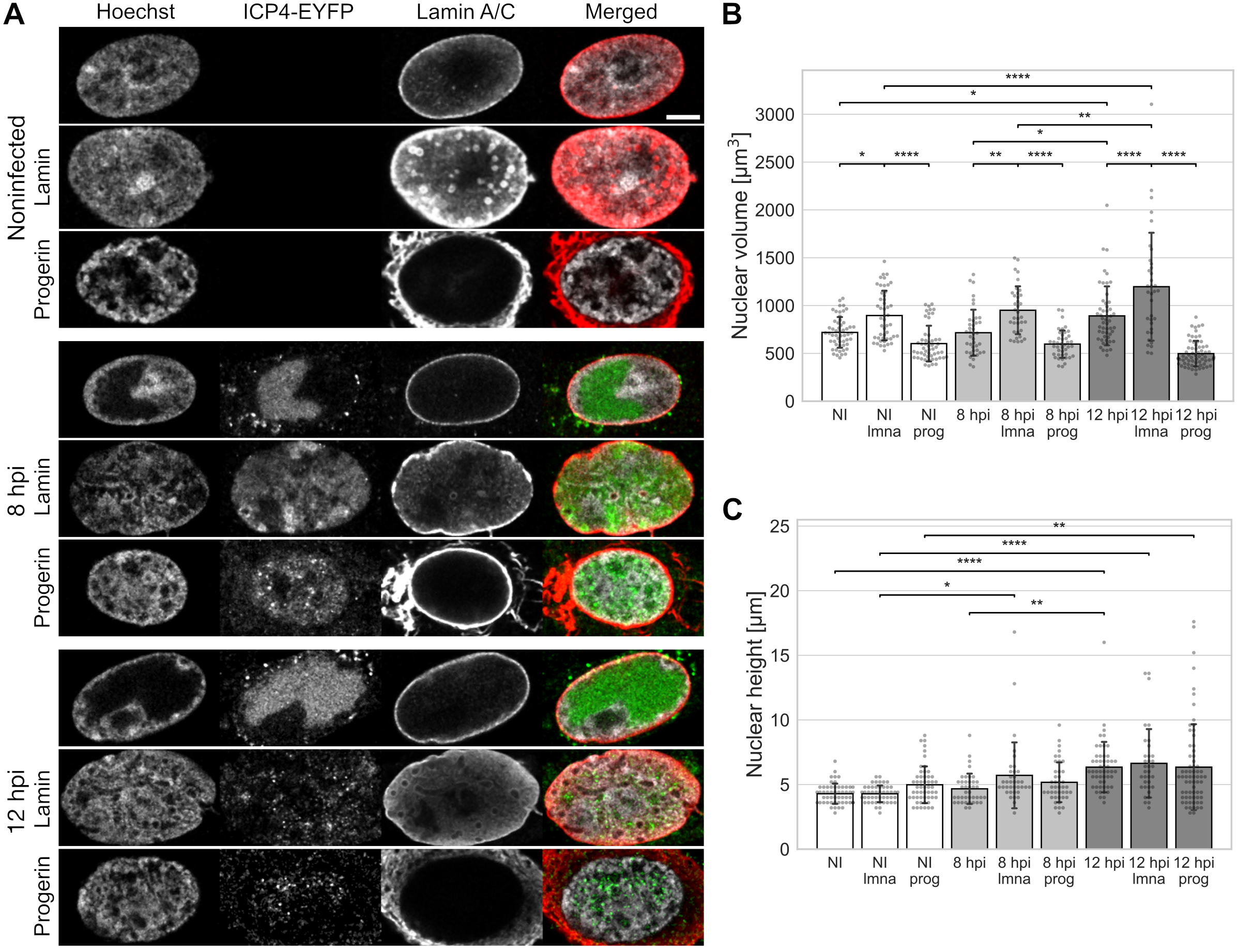

Supplement: S2 Fig — (A) Representative confocal images of noninfected and HSV-1 EYFP-ICP4-infected Vero cells at 8 and 12 hpi together with Vero cells overexpressing lamin A (lmna) and expressing progerin (prog). Lamin A/C was labeled with lamin A/C antibody (red), EYFP-ICP4 (green) shows the localization of the VRC, and the chromatin was labeled with Hoechst 33342 (grey). Quantitative analysis of (B) the volume and (C) the height of the nucleus in noninfected (NI) and infected cells (n = 53, 48, 53, 40, 36, 42, 52, 35, and 67 for the bars from left to right). The error bars show the standard deviation. Statistical significance was determined using Tukey’s test, and the significance values are denoted as **** (p < 0.0001), ** (p < 0.01), or * (p < 0.05). Only values of the same time point or treatment were statistically compared. Nonsignificant differences (p ≥ 0.05) are not labeled. (TIF) [file ppat.1013873.s002.tif]

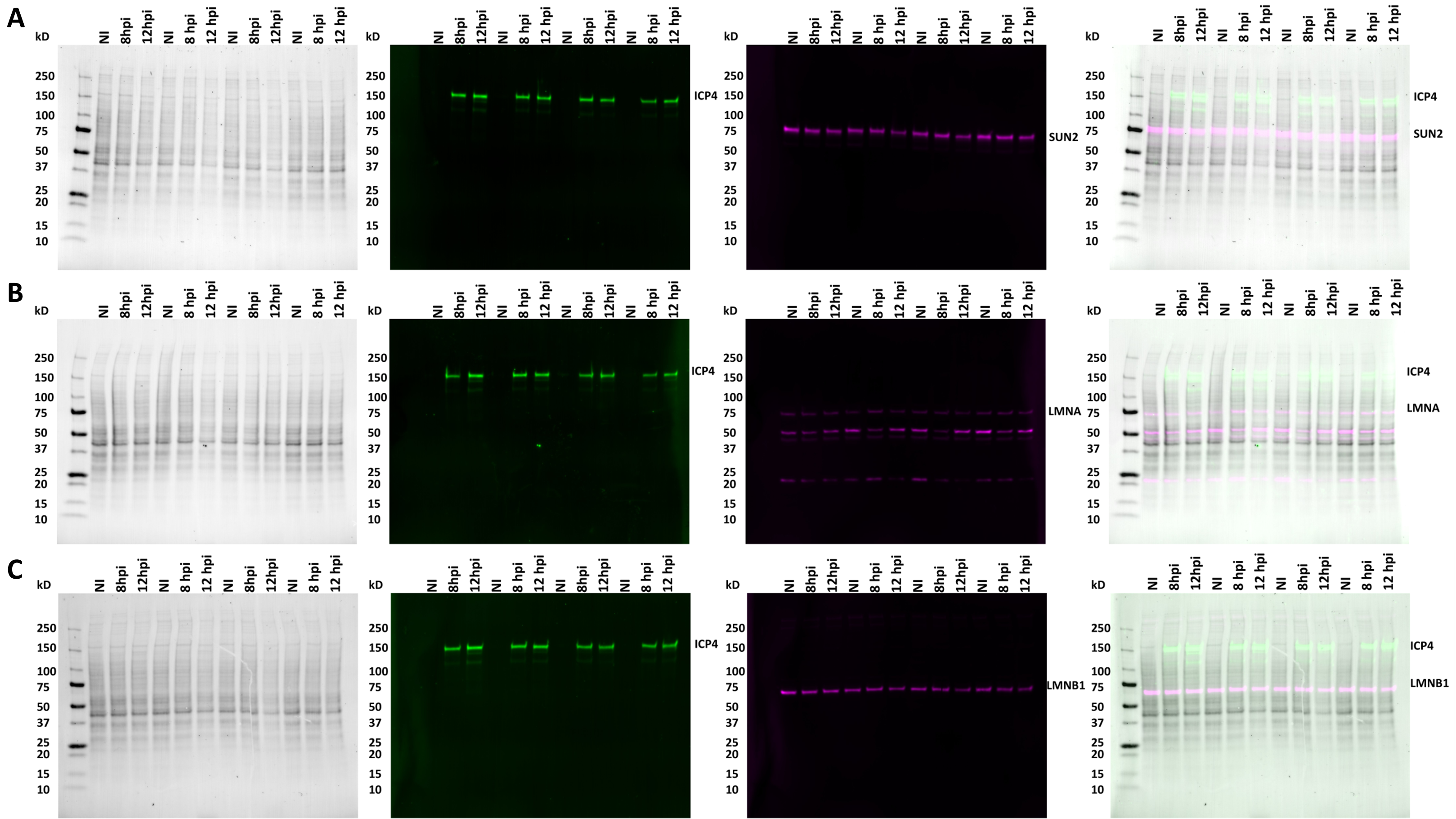

Supplement: S3 Fig — Western blot of noninfected and infected (8 and 12 hpi) Vero cell lysates were analyzed for total protein and SUN2 (A), LMNA (B), or LMNB1 (C). An antibody against ICP4 was used as a marker for viral infection. Each set of noninfected and infected lysates represents a replicate experiment (n = 4). (TIF) [file ppat.1013873.s003.tif]

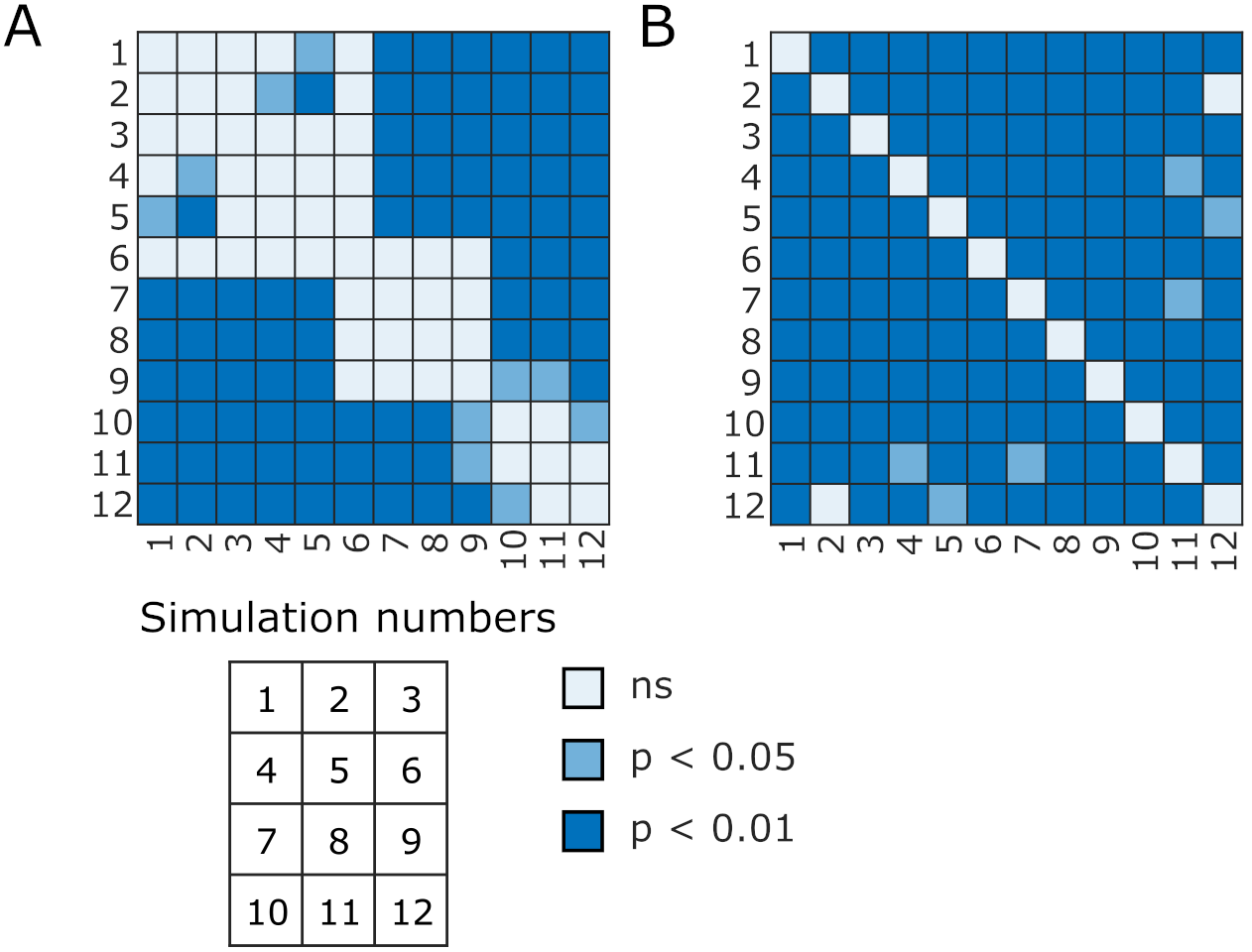

Supplement: S4 Fig — Simulation case numbers and statistical significances between simulations presented in Fig 6 for (A) Young’s moduli (6F) and (B) nuclear envelope membrane tension (6G). Statistical significances were determined using the Games-Howell test. The significance values are denoted as p < 0.01, p < 0.05, or ns (not significant). (TIF) [file ppat.1013873.s004.tif]

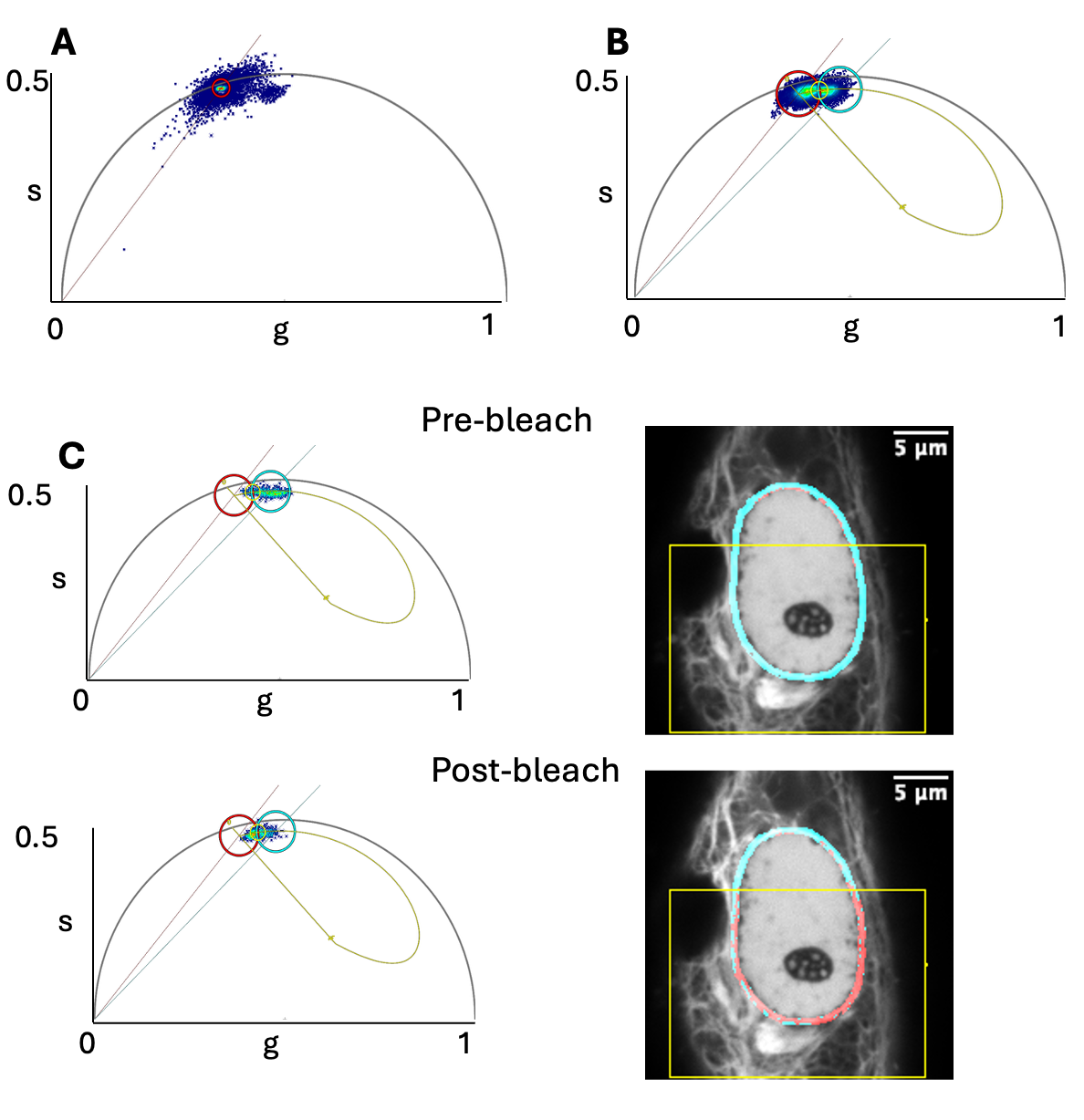

Supplement: S5 Fig — (A) Phasor plot analysis of the Nesprin TS donor fluorophore mTFP1 measured in noninfected and infected cells shows a lifetime distribution from 2.65ns to 2.71ns. (B) Phasor distribution of mTFP1 from FLIM measurement of noninfected and infected cells. The FRET trajectory was generated using LAS X (4.5.0) phasor analysis tool by setting the donor-only lifetime to 2.71 ns based on mTFP1 measurements, by locating the autofluorescence from non-transfected cells, and by adjusting the background contribution to ensure the trajectory passes through the measured phasor clouds. The division between low FRET/high tension (red phasor cursor at 2.47ns) and high FRET/low tension (cyan phasor cursor at 2.01ns) was set to 10% FRET efficiency (yellow phasor cursor). The pixels at the nuclear envelope (NE) of the measured cells were divided into low or high FRET areas according to their lifetime distribution in the phasor plot, and their relative amount was quantified. (C) An acceptor bleaching experiment to validate the Nesprin TS working in our system. FRET at the NE decreases after bleaching the acceptor from the area shown by the yellow box in a living cell. (TIF) [file ppat.1013873.s005.tif]

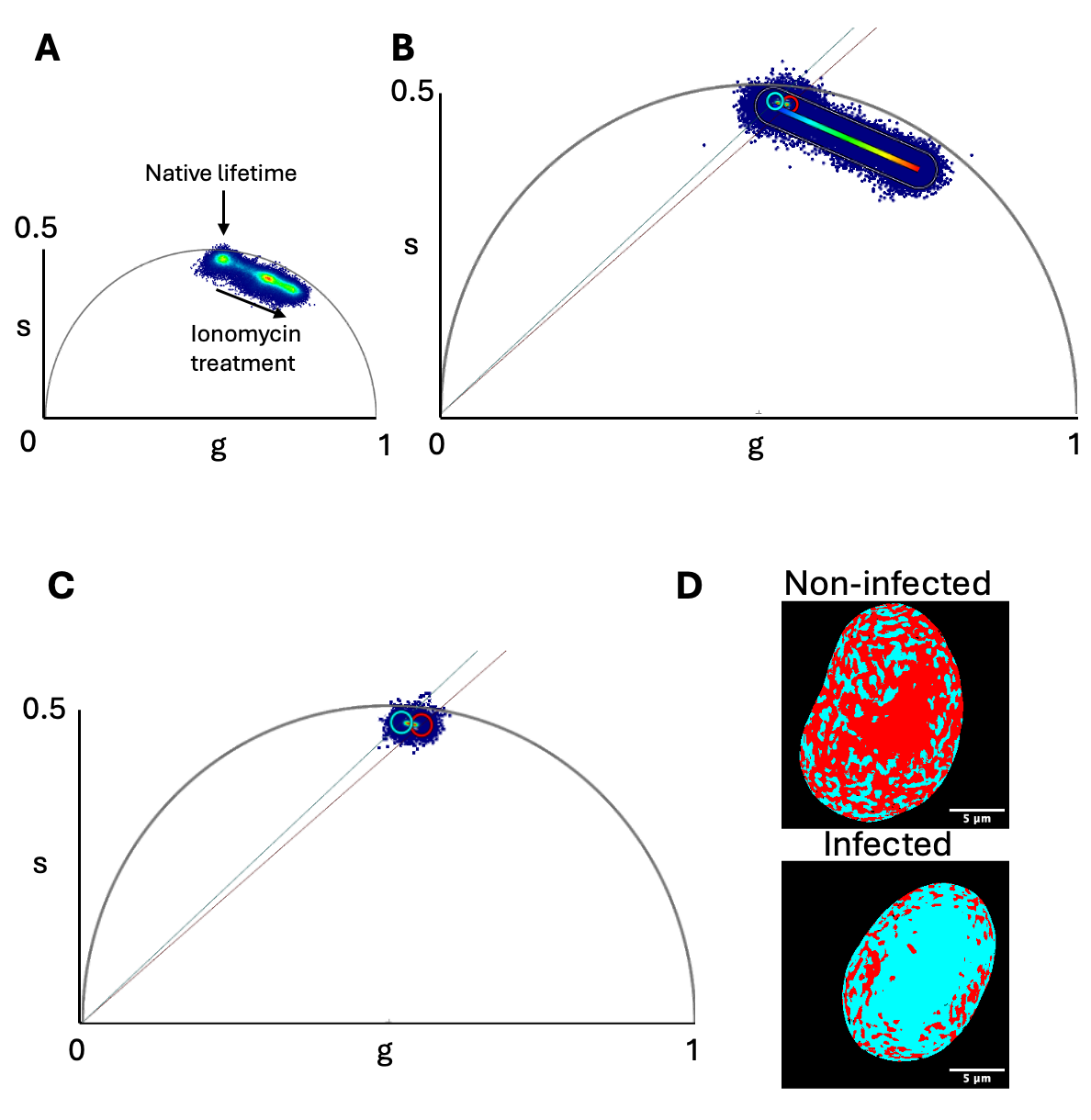

Supplement: S6 Fig — (A) Phasor plot of the phasor distribution of live FLIM imaging of 3xnls-G-Ca-FLITS transfected cells. The phasor cloud in native cell state starts approximately from s = 0.5, g = 0.5 (top-center) and moves diagonally towards down-right during ionomycin treatment, confirming the expected shorter lifetime in high calcium concentration. (B) The calibration line (rainbow), generated according to the ionomycin measurement, demonstrated longer lifetime (cyan cursor) in infected cells than in noninfected cells (red cursor). This indicated reduced calcium concentration in infected cells. (C) Phasor distribution of noninfected and infected nuclei lifetime, and their division between low calcium (longer lifetime cyan phasor cursor set to 3.71 ns) and high calcium (shorter lifetime red phasor cursor set to 3.46 ns). (D) The pseudo-colored spatial distribution of pixels with high and low calcium concentration according to their lifetime distribution in the phasor plot in representative non-infected and 8 hpi nuclei. (TIF) [file ppat.1013873.s006.tif]
