# Supplementary material for: Nucleus softens during herpesvirus infection
Source: PLoS Pathog. 2026 Jan 20;22(1):e1013873. doi: 10.1371/journal.ppat.1013873 (PMC12818678; doi:10.1371/journal.ppat.1013873)
Supplement: S1 Text — A detailed description of the model assumptions, equations, solution method, atomic force microscopy simulations and analysis, the fitting process, and the parameter values. (PDF) [file ppat.1013873.s013.pdf]

Supplementary Text: Model description

Tervonen *et al.*

December 22, 2024

1 Model summary

1.1 Model description

The model describes the nucleus as distinct nuclear envelope and chromatin components in three dimensions. The envelope, containing both the nuclear double membrane and the nuclear lamina, was triangulated into a viscoelastic shell and the chromatin was modeled by 46 self-avoiding polymer chains. Further, some chromatin vertices were connected to lamina vertices to describe the lamina-associated domain (LAD) connections between the chromatin and the lamina. In addition, interactions within different parts of a chromosome and between different chromosomes were connected by crosslinks, depicting, for example, the topologically associating domain or HP1α interactions within the chromatin mesh [1].

A nucleus shell was initialized as a spherical shell without chromatin. Next, the spherical nucleus was squeezed between two surfaces to describe the flattened nuclei of an adherent cell, and the chromatin was added inside. A spherical surface with a large radius on top was used to describe the cytoskeletal actin cap, and a planar surface below to describe the surface to which the cell is attached.

The HSV-1 viral replication compartment (VRC) was assumed to be a single coherent entity within the nucleus and was described similar to the nuclear envelope as a triangulated shape. The VRC interacted with the chromatin and the envelope via repulsive interactions.

In the atomic force microscope (AFM) simulations, the AFM cantilever was described by two points connected by a spring. The top point was moved downwards with a constant pace, and the bottom point, describing the spherical cantilever tip, contacted the nucleus.

The model was implemented using the Julia programming language (v1.10) and is available here: github.com/atervn/nuclear_mech/ [2].

1.2 Model assumptions

The main model assumptions were as follows:

- The mechanical properties of the nucleus are dictated by the nuclear lamina, nuclear membrane, chromatin, and the outward forces – including the cytoskeleton prestress and osmotic pressure difference – affecting the nucleus.
- Viscous changes in the nuclear structures occur in a time scale too slow to affect the fast AFM measurement.
- The VRC is soft and does not affect the AFM measurement

1.3 Main equations and model solution

Forces described various interactions in the model. The movements of envelope, chromatin, and VRC vertices were solved by balancing the friction and these forces and solving the overdamped equations of motion for each vertex:

$$\begin{gathered}\gamma\vec{v}_i(t) + \sum_j c_{ij}(\vec{v}_i(t) - \vec{v}_j(t)) = \vec{F}_{i,total}(t) + \eta(t) \\ \frac{d\vec{r}_i(t)}{dt} = \vec{v}_i\end{gathered} \quad , \tag{1}$$

where γ is the friction constant (kg/s), $\vec{v}_i$ is the velocity of vertex i (m/s), c_{ij} is the friction constant between vertices i and j (kg/s), $\vec{r}_i$ is the position of vertex i (m), t is time (s), $\vec{F}_{i,tot}$ is the total force acting on vertex i (N), and η is the random thermal force (N).

For the nuclear envelope shell vertices, the total force was given by

$$\vec{F}_{i,total} = \vec{F}_{i,lamina} + \vec{F}_{i,membrane} + \vec{F}_{i,outward} + \vec{F}_{i,bending} + \vec{F}_{i,lad} + \vec{F}_{i,repulsion}, \tag{2}$$

where $\vec{F}_{i,lamina}$ is the elastic force within the lamina between vertex i and neighboring vertices (N), $\vec{F}_{i,membrane}$ is the nuclear membrane force (N), $\vec{F}_{i,outward}$ describes the outward forces affecting the nucleus, including the cytoskeletal prestress and osmotic pressure difference (N), $\vec{F}_{i,bending}$ is the nuclear envelope bending force (N), $\vec{F}_{i,lad}$ is the force from the LADs between envelope vertex i and a chromatin vertex (N), and $\vec{F}_{i,repulsion}$ is the repulsive force between the envelope and chromatin vertices (N) to prevent chromatin movement through the envelope. In the case of adherent nuclei, a cellular force $\vec{F}_{i,cellular}$ is further included to keep to nucleus flattened (N). In the AFM simulations, an additional $\vec{F}_{i,afm}$ force component was included for the vertices in contact with the AFM tip (N).

For the chromatin polymer vertices, the total force is calculated

$$\vec{F}_{s,total} = \vec{F}_{s,linear} + \vec{F}_{s,bending} + \vec{F}_{s,crosslink} + \vec{F}_{s,lad} + \vec{F}_{s,exclusion} + \vec{F}_{s,repulsion}, \tag{3}$$

where $\vec{F}_{s,linear}$ is the elastic force between vertex i and neighboring vertices (N), $\vec{F}_{s,bending}$ is the polymer bending force (N), $\vec{F}_{s,crosslink}$ is the elastic crosslink force between vertices i with another chromatin vertex (N), $\vec{F}_{s,lad}$ is the force from the LADs between chromatin vertex i and an envelope vertex (N), $\vec{F}_{s,exclusion}$ is the exclusion force between chromatin vertices (N), and $\vec{F}_{s,repulsion}$ is the repulsive force between the chromatin and envelope vertices (N).

Finally, for the VRC vertices, the total force is calculated

$$\vec{F}_{i,total} = \vec{F}_{i,elastic} + \vec{F}_{i,pressure} + \vec{F}_{i,bending} + \vec{F}_{i,chroRepulsion} + \vec{F}_{i,enveRepulsion}, \tag{4}$$

where $\vec{F}_{i,elastic}$ is the elastic force that keeps the vertices of the VRC at uniform distances from each other (N), $\vec{F}_{i,pressure}$ is the growth pressure and volume force (N), $\vec{F}_{i,bending}$ is the bending force to produce smooth surfaces (N), and $\vec{F}_{i,chroRepulsion}$ and $\vec{F}_{i,enveRepulsion}$ are the repulsion forces between the VRC surface and the chromatin and envelope, respectively (N).

Equation 1 forms a linear system that can be written in the matrix form as

$$\mathbf{\Gamma}\vec{v}(t) = \vec{F}(t), \tag{5}$$

where matrix $\mathbf{\Gamma}$ contains the friction terms and is sparse and symmetric, $\vec{v}$ is the vector of vertex velocities, and $\vec{F}$ is the vector of vertex total forces. Since $\mathbf{\Gamma}$ is positive definite, conjugate gradient method can be used to solve the linear system. We used incomplete LU preconditioning. Afterwards, the solved vertex velocities were used to move the vertex positions using the forward Euler method:

$$\vec{r}_i(t + \Delta t) = \vec{r}_i(t) + \vec{v}_i(t)\Delta t, \tag{6}$$

where Δt is the time step (s). Further limitations are set for the movement of the vertices to improve stability. If any of the vertices move more than a given maximum movement distance, i.e., $\vec{v}_i(t)\Delta t > d_{max}$, the time step Δt is halved, and the movements are recalculated using conjugate gradient method for all vertices. The time step is only taken after all vertex movements are smaller than d_{max}. The same value of Δt is used by default for the next time step, however, if all vertex movements are below a given minimum movement distance, i.e., $\vec{v}_i(t)\Delta t < d_{min}$, the time step value is doubled.

Since there are friction terms between some envelope and chromatin vertices, they are solved using the same friction matrix. However, since there are no friction interactions between the VRC vertices and those of the envelope or chromatin, their movement is solved separately to reduce computational requirements.

1.4 Model creation and simulations

An icosahedron with the required radius was first defined to construct the initial triangulated spherical envelope. Next, each of the triangles formed on the surface was subdivided into four smaller ones by dividing each edge between the vertices by new vertices and connecting these vertices with new edges. Finally, the new vertices were moved outward to be at the distance of the nuclear radius from the center. The subdivision step was done four times to obtain a sufficient surface resolution. The nuclear shell was flattened between two surfaces to obtain the required volume and height before adding chromatin.

Since chromosomes are usually separated into chromosome regions with only a small amount of overlap [3], random regions of the envelope shell surface were designated for each of the 46 chromosome polymers. Each chromosome consisted of 256 vertices, each corresponding to approximately 0.5 Mbp [4]. Each chromosome was defined by a random walk with the step size of the vertex-vertex rest length d_{chro} (m). During this process, there were specific requirements for each step in the walk and the initial vertex. All vertices (1) must be within the nuclear envelope, (2) must be within a defined maximum distance from the center point of the envelope region of its chromosome, (3) cannot be closer than repulsion distance d_{rep} to any other chromatin vertices or the envelope vertices.

We estimated that there are between 5 and 15 LAD regions for each chromosome (based on the number of large continuous LAD regions in chromosomes in [5], [6], and [7]), that have a minimum distance of 10 vertices between them. These chromatin LAD vertices were then connected with random lamina vertices within the designated shell area for that chromosome. These connections were assumed to be permanent for the duration of the simulation.

Crosslinks were dynamically formed between vertices close to each other, and existing ones were broken during the simulation with given probabilities. A vertex formed a crosslink with the nearest of the neighboring vertices that are within a defined maximum crosslinking distance with the probability of $\rho_{cl,form}\Delta t$, where $\rho_{cl,form}$ is the crosslinking probability (1/s). In addition, two vertices cannot be connected if they are within the same chromosome and their distance along the chromatin is less than a given minimum limit. Existing crosslinks are broken with the probability of $\rho_{cl,break}\Delta t$, where $\rho_{cl,break}$ is the crosslinking probability (1/s). A vertex can only be a part of a single crosslink, and an LAD vertex cannot be crosslinked.

After the adherent nucleus formed initially, the system was relaxed by running the simulation for a time to allow the LAD connections to pull the chromatin close to the envelope. In addition, a long simulation was required to form the crosslinks to obtain a balanced number of crosslinks.

Finally, the VRC was created for the infected simulations. This is initialized by defining an icosahedron in the center of the nucleus with an initial size of 0.1 times the nuclear-free radius. The initial icosahedron is subdivided two times by a process similar to that of the nuclear envelope. To obtain a VRC of the required size, a simulation is run with a positive growth pressure for the VRC. During the growth, the VRC mesh is subdivided every time more than half of the mesh triangles have an area that exceeds the average initial area to retain good surface resolution. The growth stops when the required VRC volume is reached.

AFM simulations are finally run on the noninfected and infected nuclei to replicate the experimental measurements. Since we assume that the VRC is soft and thus would have little effect on the AFM results, we remove the VRC from the nuclei for the infected AFM simulation. The chromatin is still marginalized in these simulations compared to the noninfected nuclei. The creation of the nuclei for the simulations is summarized in Fig 1.

2 Friction terms

The friction terms in Equation 1 occur between pairs of vertices, and when paired with the corresponding spring force (described in the Force terms section), they form a Kelvin-Voigt element. The friction terms were included for the interactions between neighboring lamina vertices, in the LAD interactions between the lamina and chromatin vertices, as well as between the neighboring chromatin vertices. The respective friction constant for these interactions were c_{lam}, c_{lad}, and c_{chro} (kg/s).

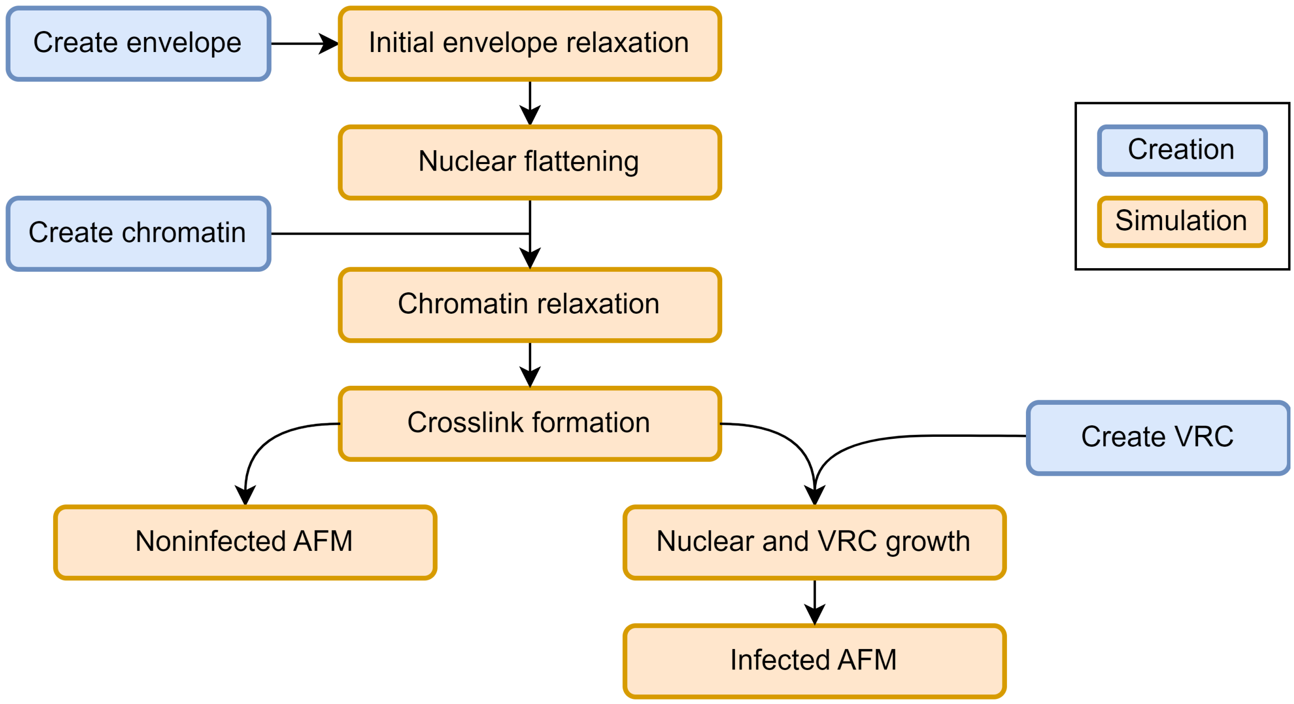

Figure 1: The process of creating a nucleus and simulating the AFM measurement.

3 Force terms

3.1 Lamina forces

The triangulated mesh of the envelope represented the lamina and its mechanical properties are incorporated as linear springs between the mesh vertices. The force was calculated as

$$\vec{F}_{i,lamina} = \sum_{j} -k_{lam} \left(r_{i,j} - l_{lam,0}\right) \hat{r}_{i,j}, \tag{7}$$

where k_{lam} is the lamina stiffness (kg/s^2), $r_{i,j} = \|\vec{r}_j - \vec{r}_i\|$ is the distance between vertices i and j (m), $l_{lam,0}$ is the rest length of the lamina connection (m), $\hat{r}_{i,j} = (\vec{r}_j - \vec{r}_i)/r_{i,j}$ is the unit vector between vertices i and j, and index j goes through all the neighbors connected to vertex i.

3.2 Membrane forces

The membrane force prevents larger changes in the local area and global of the nuclear membrane. The force on vertex i is the sum of local and global forces, or $\vec{F}_{i,area} = \vec{F}_{i,localArea} + \vec{F}_{i,globalArea}$. The local area is calculated in relation to the neighboring triangles $T_{i,j,k}$ as

$$\vec{F}_{i,localArea} = \sum_{T_{i,j,k}} k_{localArea} \frac{A_{i,j,k} - A_{i,j,k,0}}{r_{i,C}^2 + r_{j,C}^2 + r_{k,C}^2} \vec{r}_{i,C}, \tag{8}$$

where $k_{localArea}$ is the local area modulus (N/m), $A_{i,j,k}$ and $A_{i,j,k,0}$ are the current and normal area of triangle $T_{i,i,k}$ (m^2), respectively, $r_{i,C}$, $r_{j,C}$, and $r_{k,C}$ are the distance of the respective triangle corner from the triangle centroid C, and $\vec{r}_{i,C}$ is the vector between vertex i and the centroid of triangle $T_{i,j,k}$ [8]. The centroid is calculated as the mean of the triangle vertex coordinates. The global area force is calculated as

$$\vec{F}_{i,globalArea} = \sum_{T_{i,j,k}} k_{globalArea} \frac{A_{nucleus} - A_{nucleus,0}}{A_{nucleus,0}} \frac{A_{i,j,k}}{r_{i,C}^2 + r_{j,C}^2 + r_{k,C}^2} \vec{r}_{i,C}, \tag{9}$$

where $k_{globalArea}$ is the global area modulus (N/m), $A_{nucleus}$ and $A_{nucleus,0}$ are the current and normal nuclear areas (m^2), respectively [8].

3.3 Outward forces

The outward forces combine the effect of the intranuclear osmotic pressure and the cytoskeletal prestress on the nucleus. In addition, a small bulk volume force was included to resist too-large reductions in nuclear volume. The total outward force was calculated as

$$\vec{F}_{i,outward} = (P_{outward} + P_{bulk})A_i\hat{n}_i, \tag{10}$$

where $P_{outward}$ is the outward force described by a pressure (Pa),P_{bulk} is the bulk volume pressure, A_i is the surface area related to vertex i (m^2), and $\hat{n}_i$ is the surface normal at vertex i. The bulk volume pressure is calculated as $P_{bulk} = -K_{bulk}\log_1 0(V/V_0)$, where K_{bulk} is the bulk modulus (Pa), V is the current nuclear volume (m^3), and V_0 is the normal nuclear volume (m^3).

3.4 Lamina bending forces

To maintain a curvature of the nuclear envelope, a bending force was introduced between two triangles with a shared edge between them as a hinge. This is described in Fig. 2 between triangles $T_{i,j,k}$ and $T_{j,k,l}$ that share the edge $E_{j,k}$ between vertices j and k.

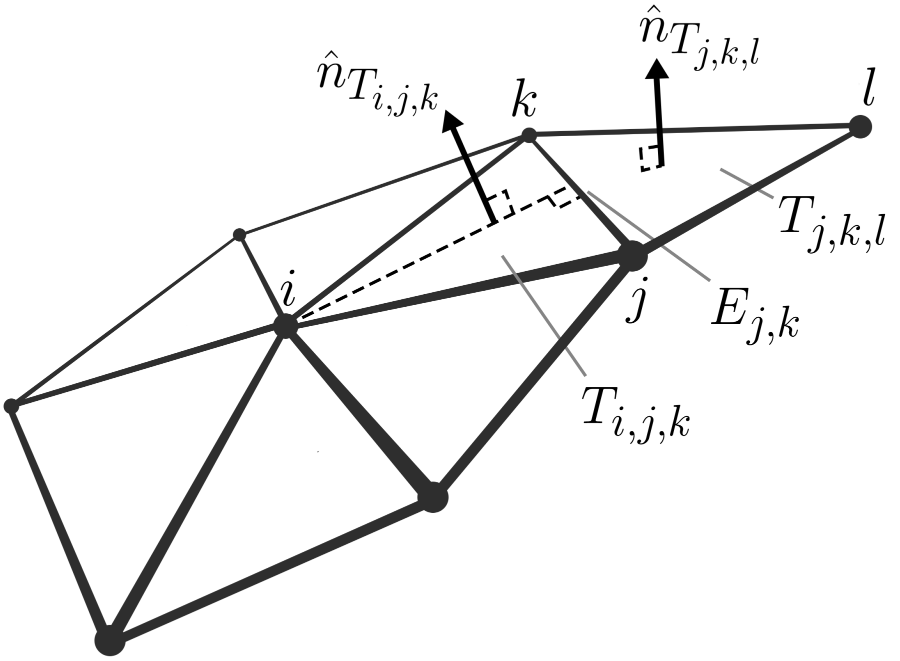

Figure 2: Test caption

The bending moment in the case described in Fig. 2 over the edge $E_{j,k}$ was calculated as

$$M_{j,k} = k_{lamBending}\sin\left(\theta_{j,k} - \theta_0\right), \tag{11}$$

where $k_{lamBending}$ is the bending stiffness (Nm), $\theta_{j,k}$ is the angle between the triangle normal vectors $\hat{n}_{T_{i,j,k}}$ and $\hat{n}_{T_{j,k,l}}$, i.e. $\theta_{j,k} = \cos^{-1}\left(\hat{n}_{T_{j,k,l}} \cdot \hat{n}_{T_{j,k,l}}\right)$ (rad), and θ_0 is the normal angle (rad). The bending force on vertex i from the moment on the edge between vertices j and k is calculated as

$$\vec{F}_{i,E_{j,k},bending} = \frac{M_{j,k}}{l_{i,E_{j,k}}}\hat{n}_{T_{i,j,k}}, \tag{12}$$

where $l_{i,E_{j,k}}$ is the shortest distance between vertex i and edge $E_{j,k}$.

Moreover, the total force for each pair of triangles and their shared edge must be zero to conserve momentum. Therefore, an additional force acts on each of the hinge vertices j and k with the magnitude of

$$-\frac{1}{2}\left(F_{i,E_{j,k},bending} + F_{l,E_{j,k},bending}\right). \tag{13}$$

The full bending force acting on a vertex is thus a sum of the different forces from all the two triangle systems in which it is a component.

3.5 Lamina-associated domain (LAD) forces

The LAD connections between the random envelope and chromatin vertices are described as linear springs, and the forces on the envelope vertex i and chromatin vertex s are thus calculated, respectively, as

$$\vec{F}_{i,lad} = \sum_{j} -k_{lad}\left(r_{i,s} - l_{lad,0}\right)\hat{r}_{i,s} \tag{14}$$

and

$$\vec{F}_{s,lad} = \sum_{j} -k_{lad}\left(r_{s,i} - l_{lad,0}\right)\hat{r}_{s,i}, \tag{15}$$

where k_{lad} is the lad strength (kg/s^2).

3.6 Repulsion forces between envelope and chromatin

A repulsive force between the chromatin polymer and the envelope shell was implemented to prevent the chromatin movement out of the nucleus. This was done by finding the closest point of the envelope for each chromatin vertex according to the algorithm by [9]. The closest point was either on the vertex of a triangle, on its edge, or on its surface. The force on the polymer vertex s can then be calculated as

$$\vec{F}_{s,repulsion} = \begin{cases} -k_{rep}\left(d_{rep} - \|\vec{r}_s - \vec{r}_{e,s}\|\right)\frac{\vec{r}_s - \vec{r}_{e,s}}{\|\vec{r}_s - \vec{r}_{e,s}\|} & \text{if } \|\vec{r}_s - \vec{r}_{e,s}\| < d_{rep} \\ 0 & \text{if } \|\vec{r}_s - \vec{r}_{e,s}\| \geq d_{rep} \\ -0.5k_{rep}\frac{\vec{r}_s - \vec{r}_{e,s}}{\|\vec{r}_s - \vec{r}_{e,s}\|} & \text{if } \vec{r}_s \text{ outside} \end{cases}, \tag{16}$$

where k_{rep} is the repulsion constant (kg/s^2), d_{rep} is the repulsion distance (m), $\vec{r}_{e,s}$ is the envelope point closest to the chromatin vertex s (m). The third case is for the situation where the chromatin has moved outside the envelope, e.g., due to a too-large time step, and has to be pushed back inside. A dot product between the force unit vector and the surface normal is calculated to check if a chromatin point is outside. If the dot product is positive, then the vectors are at most right angle to each other, indicating that the chromatin vertex is outside the envelope.

In addition to the force on the chromatin vertex, the repulsion also affects the envelope with a force depending on whether the chromatin vertex is closest to the triangle vertex, edge, or surface. These forces are summed over all the repulsion interactions in which an envelope vertex is a part. In the first case, the repulsive force on the envelope vertex is opposite to that on the chromatin vertex, i.e

$$\vec{F}_{i,repulsion} = -F_{s,repulsion} \tag{17}$$

In the second case, where the closest point is on the edge between two envelope vertices, the force acts on the two vertices i and j with forces

$$\begin{aligned} \vec{F}_{i,repulsion} &= -w_i F_{s,repulsion} \\ \vec{F}_{j,repulsion} &= -w_j F_{s,repulsion} \end{aligned}, \tag{18}$$

where w_i and w_j are the weights calculated based on linear interpolation from the equation for the x-coordinates (assuming that $x_i \neq x_j$):

$$\begin{aligned} w_i &= \frac{x_{e,s} - x_i}{x_j - x_i} \\ w_j &= 1 - w_i \end{aligned}. \tag{19}$$

In the third case, with the triangle surface itself being closest to the chromatin vertex, the force is divided between all three vertices:

$$\begin{aligned} \vec{F}_{i,repulsion} &= -w_i \vec{F}_{s,repulsion} \\ \vec{F}_{j,repulsion} &= -w_j \vec{F}_{s,repulsion}, \\ \vec{F}_{k,repulsion} &= -w_k \vec{F}_{s,repulsion} \end{aligned} \tag{20}$$

where w_i, w_j, and w_k are the barycentric weights for the respective triangle vertex and are defined as

$$\begin{aligned} w_i &= \frac{1}{2A_{ijk}}\|\vec{r}_{e,s} - \vec{r}_j\| \cdot \|\vec{r}_{e,s} - \vec{r}_k\| \\ w_j &= \frac{1}{2A_{ijk}}\|\vec{r}_{e,s} - \vec{r}_i\| \cdot \|\vec{r}_{e,s} - \vec{r}_k\| \\ w_k &= \frac{1}{2A_{ijk}}\|\vec{r}_{e,s} - \vec{r}_i\| \cdot \|\vec{r}_{e,s} - \vec{r}_j\| \\ A_{ijk} &= \frac{1}{2}\|\vec{r}_i - \vec{r}_j\| \cdot \|\vec{r}_i - \vec{r}_k\| \end{aligned}, \tag{21}$$

where A_{ijk} is the area of the triangle defined by vertices i, j, and k (m^2).

3.7 Chromatin linear forces

Within a chromosome polymer, the monomer vertices were connected to their linear neighbors with elastic interactions. Thus, other than the chromosome ends, each vertex had two neighbors. These linear forces were calculated with the equation

$$\vec{F}_{s,linear} = \begin{cases} -k_{chro}\left(r_{s,s+1} - d_{chro}\right)\hat{r}_{s,s+1} & \text{if } s = 1 \\ -k_{chro}\left(r_{s,s-1} - d_{chro}\right)\hat{r}_{s,s-1} & \text{if } s = n_{chro} \\ -k_{chro}\left(\left(r_{s,s+1} - d_{chro}\right)\hat{r}_{s,s+1} + \left(r_{s,s-1} - d_{chro}\right)\hat{r}_{s,s-1}\right) & \text{otherwise} \end{cases}, \tag{22}$$

where k_{chro} is the linear chromatin stiffness ($\mathrm{kg/s^2}$), d_{chro} is the normal chromatin vertex separation (m), and n_{chro} is the number of vertices per chromosome.

3.8 Chromatin bending forces

A bending force acted on the chromatin monomers when the bend on the neighboring monomers differed from the normal polymer angle $\theta_{0,chro}$. The forces from the neighboring vertices t were calculated as

$$\vec{F}_{s,bending} = \sum_t -\frac{k_{chroBending}}{r_{s,t}}\left(\theta_{s,t,r} - \theta_{chro,0}\right)\hat{r}_{s,t,r}, \tag{23}$$

where $k_{chroBending}$ is the bending stiffness (Nm), $\theta_{s,t,r}$ is the angle in between three neighboring vertices s, t, and r, where t is the hinge, $\theta_{chro,0}$ is the normal chromatin angle, and $\hat{r}_{s,t,r}$ is the unit vector that is in the plane formed by the three vertices and perpendicular to the vector $\vec{r}_{s,t}$ and calculate as

$$\hat{r}_{s,t,r} = \frac{\vec{r}_{t,s} \times \left(\vec{r}_{t,s} \times \vec{r}_{t,r}\right)}{\|\vec{r}_{t,s} \times \left(\vec{r}_{t,s} \times \vec{r}_{t,r}\right)\|}. \tag{24}$$

3.9 Crosslink forces

The crosslinks were described by simple springs, and the force between vertex s and its crosslink pair t was calculated as

$$\vec{F}_{s,crosslink} = -k_{crosslink}\left(r_{s,t} - d_{crosslink}\right)\hat{r}_{s,t}, \tag{25}$$

where $k_{crosslink}$ is the crosslink spring constant ($\mathrm{kg/s^2}$) and $d_{crosslink}$ is the crosslink rest length (m).

3.10 Chromatin exclusion

Chromatin exclusion is implemented by adding a repulsive force between the chromatin vertices. The repulsion occurs only when the vertices are closer than a repulsion distance d_{rep}, and is described by the equation

$$\vec{F}_{s,exclusion} = \sum_t \begin{cases} -k_{rep}\left(r_{s,t} - d_{rep}\right)\hat{r}_{s,t} & \text{if } r_{s,t} < d_{rep} \\ 0 & \text{if } r_{s,t} \geq d_{rep} \end{cases}, \tag{26}$$

where $r_{s,t}$ is the distance between chromatin vertices s and t (m).

3.11 Cellular forces

The cellular forces described the flattening of the nucleus between the basal side of the cell in contact with the substrate below and the apical actin cap in tension pushing down. Therefore, the cytoskeleton force was calculated as

$$\vec{F}_{i,cellular} = \vec{F}_{i,basal} + \vec{F}_{i,apical}. \tag{27}$$

The force from the interactions on the basal side ($\vec{F}_{i,basal}$) was calculated assuming that there is a plane with a specified z-coordinate below the nucleus that repels it upwards:

$$\vec{F}_{i,basal} = \begin{cases} -k_{rep}(r_{i,b} - d_{rep})\hat{n}_z & \text{if } 0 \le z_i - z_b \le d_{rep} \\ k_{rep}\hat{n}_z & \text{if } z_b - z_i < 0 \\ 0 & \text{otherwise} \end{cases}, \tag{28}$$

where $r_{i,b}$ is the distance of vertex i from the basal plane, $\hat{n}_z$ is an unit vector in positive z-direction, z_i and z_b are the z-coordinates of vertex i and the basal plane, respectively.

The apical force $\vec{F}_{i,apical}$ assumed that a curved surface, depicting the cell surface, was pushing down on the nucleus. The curved surface was defined by a sphere with a center point $\vec{r}_a$ and radius R_a. The force was calculated as

$$\vec{F}_{i,apical} = \begin{cases} -k_{rep}(R_a - r_{i,a} - d_{rep})\hat{r}_{i,a} & \text{if } 0 \le R_a - \|\vec{r}_a - \vec{r}_i\| \le d_{rep} \\ -k_{rep}\hat{r}_{i,a} & \text{if } R_a - \|\vec{r}_a - \vec{r}_i\| < 0 \\ 0 & \text{otherwise} \end{cases}. \tag{29}$$

Since the nuclear envelope was initially built as a sphere, it is flattened by moving the center of the apical sphere downwards until the required nuclear height is achieved.

3.12 Atomic force microscope (AFM) forces

The AFM forces only affected vertices in contact with the AFM tip. The tip repulsed the vertices similarly, e.g., to the cellular forces. The AFM forces were calculated as:

$$\vec{F}_{i,afm} = \begin{cases} -k_{afmRep}(r_{i,tip} - d_{rep})\hat{r}_{i,tip} & \text{if } 0 \le r_{i,tip} \le d_{rep} \\ -k_{afmRep}\hat{r}_{i,tip} & \text{if } r_{i,tip} < 0 \\ 0 & \text{otherwise} \end{cases}, \tag{30}$$

where k_{afmRep} is the repulsive force constant between the nucleus and the AFM tip, and $r_{i,tip}$ is the distance between vertex i and the tip surface, calculated as $r_{i,tip} = \|\vec{r}_{tip} - \vec{r}_i\| - R_{tip}$, where $\vec{r}_{tip}$ is the AFM tip point position and R_{tip} is the radius of the spherical tip (m).

3.13 Random thermal forces

The thermal motion of the envelope and chromatin vertices was modeled using a random force based on the Langevin equation:

$$\eta(t) = \sqrt{2k_B T \gamma}\frac{d\xi(t)}{dt}, \tag{31}$$

where k_B is the Boltzmann constant (1.38×10^{-23} m^2 kg/(s^2 K)), T is absolute temperature (K), and ξ is a random variable at time t from Gaussian distribution with zero mean and variance of 1 [10].

3.14 Viral replication compartment (VRC) forces

The force components for the VRC largely mimicked those of the nuclear envelope. Therefore, the elastic force is calculated using Eq. 7. However, since the aim of the elastic force was only to retain the uniform vertex spacing, the normal length was defined as the average VRC edge length for each time step. The bending force was calculated with the envelope in Eq. 12 and 13. Chromatin repulsion force was solved between chromatin and envelope using Eq. 16. These equations were also used with the envelope repulsion by assuming the envelope vertices as the vertices in the interaction. The growth pressure force either aimed to increase the size of the VRC or retain current volume, depending on the situation.

4 AFM cantilever and analysis

The description of the AFM cantilever with a spring attached to a spherical tip allowed the inclusion of the properties of the real AFM system used in the experiments. During the simulation, the tip pushed the nucleus, and the nucleus reciprocally pushed the tip, compressing the spring between the two cantilever points. The simulation was stopped when the cantilever spring reached the maximum force used in the experimental measurements.

The positive vertical total force components of all the envelope vertices in contact with the tip were summed to calculate the force with which the nucleus pushed upwards on the tip. This force was then used to push on the cantilever spring, compressing it. To calculate the movement of the AFM tip, the following equation was solved:

$$\begin{aligned} \gamma \vec{v}_t ip(t) + c_{afm}(\vec{v}_t ip(t) - \vec{v}_t op(t)) &= \vec{F}_{afm,total}(t) \\ \frac{d\vec{r}_t ip(t)}{dt} &= \vec{v}_t ip \end{aligned}, \tag{32}$$

where c_{afm} is the cantilever friction coefficient (kg/s) and $\vec{F}_{afm,total}(t) = \vec{F}_{cantilever}(t) + \vec{F}_{nucleus}(t)$, where $\vec{F}_{cantilever}(t)$ and $\vec{F}_{nucleus}(t)$ are the cantilever and nucleus force on the tip, respectively (N). The cantilever spring force was calculated as $\vec{F}_{cantilever} = k_{afm}\left(z_{tip,top} - d_{cantilever}\right)$, where k_{afm} is the cantilever spring constant (kg/s^2), $z_{tip,top}$ is the distance between the bottom tip point and the top cantilever point (m), and $d_{cantilever}$ is the normal spring length (m).

After the simulation, the AFM indentation force and depth values were obtained, and the stiffness was defined using the Hertz model for spherical tip:

$$F = \frac{4}{3}\frac{E}{(1-\nu^2)}\sqrt{R_{tip}}\delta^{\frac{3}{2}}, \tag{33}$$

where F is the force measured by the cantilever (N), E is the measured Young's modulus (Pa), ν is the Poisson's ratio of the nucleus (estimated 0.5), and δ is the indentation depth (m). The analysis was conducted by fitting the data to the equation to obtain E. This was done using Matlab (MathWorks Inc, version R2021a, Natick, Massachusetts).

5 Parameter values

The parameter values used in the model are based on the literature, fitted, or chosen based on preliminary simulations. The nuclear dimensions and properties are summarized in Table 1. When squeezed with the cellular forces, the initial nuclear radius was chosen to produce the experimentally observed noninfected nuclear size. The chromatin vertex-to-vertex rest length was selected to form a sufficient length of chromatin fibers within the nucleus. In addition, LAD and crosslink rest lengths, as well as repulsion distance, were chosen to equal the chromatin vertex rest length for simplicity. The normal angle between chromatin segments was determined based on the assumption that the chromatin naturally straightens. The radius for the actin cap was chosen based on preliminary simulations to produce an appropriate nuclear shape. Based on preliminary simulations, the chromatin crosslink formation

and breaking probabilities were selected to produce the 20 % of crosslinked chromatin vertices as also utilized by [1].

Table 1: Nucleus dimensions are properties

Parameter	Symbol	Value	Unit	Source
Initial nucleus radius	$r_{nucleus}$	6.1×10^{-6}	m	PS
Chromatin vertex-to-vertex rest length	d_{chro}	3×10^{-7}	m	PS
LAD rest length	d_{chro}	3×10^{-7}	m	PS
Crosslink rest length	d_{chro}	3×10^{-7}	m	PS
Repulsion distance	d_{rep}	3×10^{-7}	m	PS
Chromatin normal angle	$\theta_{chro,0}$	180	deg	PS
Actin cap radius	R_a	15×10^{-6}	m	PS
Chromatin crosslink formation probability	$\rho_{cl,form}$	0.02	1/s	[1], PS
Chromatin crosslink breaking probability	$\rho_{cl,break}$	0.1	1/s	[1], PS

PS, preliminary simulations

The nuclear and VRC dimensions used in the simulations were obtained from our imaging data. The nuclear volume, height, and VRC volume data are summarized in Table 2.

Table 2: Nucleus dimensions are properties

Timepoint	Nuclear volume (μm^3)	Nuclear height (μm)	VRC volume (μm^3)
Noninfected	719	4.3	-
8 hpi	716	4.6	200
12 hpi	892	6.3	250

The mechanical parameters for the model are summarized in Table 3. The values for the lamina and chromatin (stiffnesses and bending stiffnesses) were obtained by fitting the model with experimental AFM simulations after setting the other parameter values. The obtained values for the lamina and chromatin linear stiffnesses are in a similar order of magnitude to those used by [11]. In addition, the values for the LAD and crosslink stiffnesses and the repulsion constant were assumed to equal the chromatin stiffness. The local and global compression stiffnesses were considered to be in a similar order of magnitude as those for the cell membrane [8]. Nuclear bulk modulus, excluding the effect of the chromatin, was assumed to be small due to the nuclear pores that allow water flow. The outward forces were considered to be on the scale of a hundred pascals based on [12]. The values for the frictions were chosen based on preliminary simulations to minimize the spring oscillations while minimizing their effect on the mechanics.

The mechanical properties of the VRC were not crucial for the simulations since we assumed that this low-density structure is very soft and has no effect on the AFM results. Therefore, the parameters, summarized in Table 4, are only used while marginalizing the chromatin with the growing VRC during the initial simulations before the AFM simulation. The growth pressure was chosen to grow the VRC at a stable rate.

The simulation-related parameters are summarized in Table 5. The scaling time, length, and viscosity were used to nondimensionalize the model parameters. The viscosity value for the nuclear creation was chosen based on simulation stability. For the crosslink formation, a much higher value was used to increase the time step to obtain a stable amount of crosslinks. A smaller value was used for the AFM simulations to remove the viscous effects from the measurement. The maximum and minimum step sizes for the simulation time stepping were chosen to have stable simulations and, e.g., prevent too large repulsive forces due to large spatial steps.

Parameters for the AFM cantilever are summarized in Table 6. The parameter values were based on experiments other than the cantilever friction and cantilever spring length, which were chosen based on preliminary simulations.

Table 3: Nucleus mechanical parameters

Parameter	Symbol	Value	Unit	Source
Lamina stiffness	k_{lam}	15×10^{-4}	kg/s^2	F
Lamina bending stiffness	$k_{lamBending}$	1×10^{-17}	Nm	F
Chromatin stiffness	k_{chro}	15×10^{-4}	kg/s^2	F
Chromatin bending stiffness	$k_{chroBending}$	1×10^{-18}	Nm	F
Local area compression stiffness	$k_{localArea}$	5×10^{-5}	N/m	[8]
Global area compression stiffness	$k_{globalArea}$	1×10^{-3}	N/m	[8]
Bulk modulus	k_{volume}	100	Pa	PS
Outward forces	$P_{outward}$	100	Pa	[12]
LAD stiffness	k_{lad}	15×10^{-4}	kg/s^2	F
Crosslink stiffness	$k_{crosslink}$	15×10^{-4}	kg/s^2	F
Repulsion constant	k_{rep}	15×10^{-4}	kg/s^2	F
Lamina friction	c_{lam}	1×10^{-4}	kg/s	PS
Chromatin friction	c_{chro}	1×10^{-4}	kg/s	PS
LAD friction	c_{lad}	1×10^{-4}	kg/s	PS

F, fitted; PS, preliminary simulations

Table 4: Viral replication compartment mechanical parameters

Parameter	Symbol	Value	Unit	Source
VRC stiffness	k_{vrc}	15×10^{-4}	kg/s^2	PS
VRC bending stiffness	$k_{vrcBending}$	3×10^{-17}	kg/s^2	PS
VRC growth pressure	P_{vrc}	300	Pa	PS
VRC friction	c_{vrc}	1×10^{-4}	kg/s	PS

PS, preliminary simulations

Table 5: Simulation parameters

Parameter	Symbol	Value	Unit	Source
Scaling time	$\mathbb{T}$	60	s	PS
Scaling length	$\mathbb{L}$	1×10^{-6}	m	PS
Viscosity	η	1×10^{-4}	kg/s	PS
Viscosity (crosslink formation)	η	0.1	kg/s	PS
Viscosity (AFM)	η	5×10^{-7}	kg/s	PS
Maximum vertex movement	d_{max}	2×10^{-7}	m	PS
Minimum vertex movement	d_{min}	1×10^{-7}	m	PS
Maximum time step	Δt_{max}	0.1	s	PS
Maximum time step (crosslink formation)	Δt_{max}	2	s	PS
Maximum time step (AFM)	Δt_{max}	0.001	s	PS

PS, preliminary simulations

References

[1] Amy R Strom, Ronald J Biggs, Edward J Banigan, Xiaotao Wang, Katherine Chiu, Cameron Herman, Jimena Collado, Feng Yue, Joan C Ritland Politz, Leah J Tait, David Scalzo, Agnes Telling, Mark Groudine, Clifford P Brangwynne, John F Marko, and Andrew D Stephens. HP1 is a chromatin crosslinker that controls nuclear and mitotic chromosome mechanics. *eLife*, 10: e63972, June 2021.

[2] Aapo Tervonen. Nuclear_mech v1.0.1, 2024. URL `https://zenodo.org/records/14512358`.

Table 6: AFM cantilever parameters

Parameter	Symbol	Value	Unit	Source
Tip radius	R_{tip}	3.31×10^{-6}	m	E
Cantilever spring constant	k_{afm}	0.05	kg/s^2	E
Cantilever speed	v_{afm}	1×10^{-6}	m/s	E
Cantilever friction coefficient	c_{afm}	10	kg/s	PS
Cantilever spring length	d_{cant}	10×10^{-6}	m	PS

PS, preliminary simulations, E, experimental system

[3] Thomas Cremer and Marion Cremer. Chromosome Territories. *Cold Spring Harbor Perspectives in Biology*, 2(3):a003889, March 2010.

[4] Allison Piovesan, Maria Chiara Pelleri, Francesca Antonaros, Pierluigi Strippoli, Maria Caracausi, and Lorenza Vitale. On the length, weight and GC content of the human genome. *BMC Research Notes*, 12(1):106, February 2019.

[5] Nolwenn Briand and Philippe Collas. Lamina-associated domains: peripheral matters and internal affairs. *Genome Biology*, 21(1):85, April 2020.

[6] Parisha P. Shah, Kathleen C. Keough, Ketrin Gjoni, Garrett T. Santini, Richard J. Abdill, Nadeera M. Wickramasinghe, Carolyn E. Dundes, Ashley Karnay, Angela Chen, Rachel E. A. Salomon, Patrick J. Walsh, Son C. Nguyen, Sean Whalen, Eric F. Joyce, Kyle M. Loh, Nicole Dubois, Katherine S. Pollard, and Rajan Jain. An atlas of lamina-associated chromatin across twelve human cell types reveals an intermediate chromatin subtype. *Genome Biology*, 24(1):16, January 2023.

[7] Bas van Steensel and Andrew S. Belmont. Lamina-Associated Domains: Links with Chromosome Architecture, Heterochromatin, and Gene Repression. *Cell*, 169(5):780–791, May 2017.

[8] Iveta Jančigová, Kristína Kovalčíková, Alžbeta Bohiniková, and Ivan Cimrák. Spring-network model of red blood cell: From membrane mechanics to validation. *International Journal for Numerical Methods in Fluids*, 92(10):1368–1393, 2020.

[9] David Eberly. Distance Methods. In *3D Game Engine Design: A Practical Approach to Real-Time Computer Graphics*. CRC Press, USA, 2nd edition, 2006.

[10] A. Amitai and D. Holcman. Polymer physics of nuclear organization and function. *Physics Reports*, 678:1–83, March 2017.

[11] Andrew D. Stephens, Edward J. Banigan, Stephen A. Adam, Robert D. Goldman, and John F. Marko. Chromatin and lamin A determine two different mechanical response regimes of the cell nucleus. *Molecular Biology of the Cell*, 28(14):1984–1996, July 2017.

[12] Dan Deviri and Samuel A. Safran. Balance of osmotic pressures determines the nuclear-to-cytoplasmic volume ratio of the cell. *Proceedings of the National Academy of Sciences*, 119(21): e2118301119, May 2022.
